# Transcriptomic Landscape of Lower Grade Glioma Based on Age-Related Non-Silent Somatic Mutations

**DOI:** 10.3390/curroncol28030210

**Published:** 2021-06-19

**Authors:** YoungJoon Park, JeongMan Park, Ju Won Ahn, Jeong Min Sim, Su Jung Kang, Suwan Kim, So Jung Hwang, Song-Hee Han, Kyoung Su Sung, Jaejoon Lim

**Affiliations:** 1 Institute Department of Biomedical Science, College of Life Science, CHA University, Seongnam 13488, Korea; yjparkep@chauniv.ac.kr (Y.P.); jungman.park@chauniv.ac.kr (J.P.); eugene@chauniv.ac.kr (J.W.A.); sujung_k@chauniv.ac.kr (S.J.K.); 2 Department of Neurosurgery, Bundang CHA Medical Center, CHA University College of Medicine, Seongnam 13496, Korea; simti123@chauniv.ac.kr (J.M.S.); suwankimm@chauniv.ac.kr (S.K.); sjhwang7@chamc.co.kr (S.J.H.); 3 Global Research Supporting Center, Bundang CHA Medical Center, CHA University College of Medicine, Seongnam 13496, Korea; 4 Department of Pathology, Dong-A University College of Medicine, Dong-A University, Busan 49201, Korea; freeate@dau.ac.kr; 5 Department of Neurosurgery, Dong-A University Hospital, Dong-A University College of Medicine, Busan 49201, Korea

**Keywords:** age, glioma, mutation, TCGA, transcriptomic analysis

## Abstract

Glioma accounts for 80% of all malignant brain tumours and is the most common adult primary brain tumour. Age is an important factor affecting the development of cancer, as somatic mutations accumulate with age. Here, we aimed to analyse the significance of age-dependent non-silent somatic mutations in glioma prognosis. Histological tumour grade depends on age at diagnosis in patients with *IDH1*, *TP53*, *ATRX*, and *EGFR* mutations. Age of patients with wild-type *IDH1* and *EGFR* increased with increase in tumour grade, while the age of patients with *IDH1* or *EGFR* mutation remained constant. However, the age of patients with *EGFR* mutation was higher than that of patients with *IDH1* mutation. The hierarchical clustering of patients was dominantly separated by *IDH1* and *EGFR* mutations. Furthermore, patients with *IDH1* mutation were dominantly separated by *TP53* and *ATRX* double mutation and its double wild-type counterpart. The age of patients with *ATRX* and *TP53* mutation was lower than that of patients with wild-type *ATRX* and *TP53*. Patients with the double mutation showed poorer prognosis than those with the double wild type genotype. Unlike *IDH1* mutant, *IDH1* wild-type showed upregulation of expression of epithelial mesenchymal transition associated genes.

## 1. Introduction

Glioma, accounting for 80% of all malignant brain tumours, is the most common primary brain tumour in adults [1], and is usually treated using radiation therapy and using Temozolomide treatment after maximal safe resection [2]. Histologically, gliomas are classified as lower grade glioma (LGG) “[World Health Organization (WHO) grade 2 (G2) and 3 (G3)]” and glioblastoma (GBM) “[WHO grade 4 (G4)]”. LGG has a better prognosis than GBM, and the efficacy of treatment depends on the molecular subtype of LGG in perspective of overall survival (OS) and progression free survival. In contrast, GBM has poor prognosis, and the development of new treatment methods based on its molecular characteristics is difficult because of infiltrative and integrative characteristics of normal brain tissues [3,4].

Age is an important factor affecting the development of cancer. Most adult cancers develop exponentially with age [5], owing to the accumulation of somatic mutations [5,6,7]. However, all accumulated mutations do not contribute to cancer development. A mutation that contributes to the development of cancer is called a driver; a driver undergoes positive selection in the tissue microenvironment and leads to the development of cancer cell characteristics, such as cell growth [7,8]. There are four subtypes of glioma according to the transcriptome patterns, namely, proneural, neural, classical, and mesenchymal. The proneural subtype is associated with early onset compared with the other subtypes. The mesenchymal subtype has the worst prognosis in elderly patients [9].

Since 2016, the histological method of subtype classification has been changed to one based on molecular parameter standards, according to which, lower-grade gliomas with the *IDH1* mutation and *TP53* mutation or *ATRX* loss are classified as diffuse astrocytoma (*IDH1* mutant), gliomas with the *IDH1* mutation and 1p/19q co-deletion are classified as oligodendroglioma, and those with *IDH1* wild-type are classified as diffuse astrocytoma [10].

Previous studies have reported that the *IDH1* mutation is associated with proneural subtype of GBM which shows early onset with better prognosis [9]. GBMs with abnormal *EGFR* and *NF1* expression are classified as classical and mesenchymal subtypes [9]. In addition, the onset age of secondary glioblastoma in patients with the *IDH1* and *TP53* mutations is earlier than that of primary glioblastoma [11], and age-specific mutation frequency varies among different age groups [12]. Although, there are several studies on the relationship between age and somatic mutations, potential biological mechanisms or pathways according to transcriptomic patterns with age-related somatic mutations are not clear. In this study, we investigated the effects of age at which non-silent somatic mutations occurred on patient prognosis and analysed their transcriptomic significance and their biological mechanisms or pathways using The Cancer Genome Atlas (TCGA) transcriptomic landscape of glioma.

## 2. Results

### 2.1. Identification of Age-Related Non-Silent Mutations in Lower Grade Glioma (LGG) and Glioblastoma (GBM)

**Figure 1:**
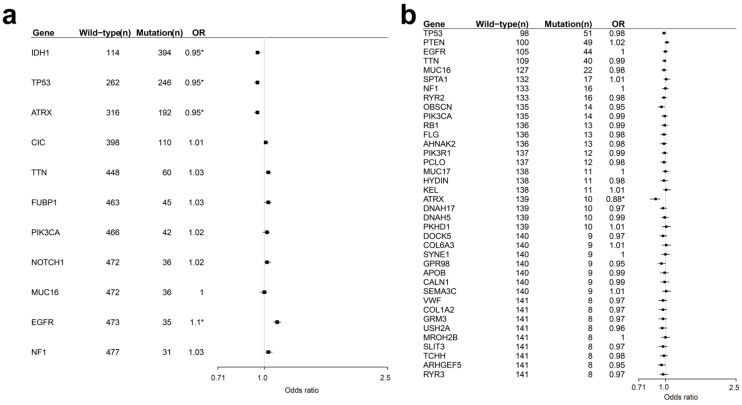
Frequencies of mutated and wild-type genes and logistic regression results showing the relationship of mutations with age in patients with lower grade glioma (**a**) and glioblastoma (**b**). Genes shown have mutation rates >5%. OR, odds ratio; *, significant.

Clinical data and non-silent somatic mutation data by multi-centre mutation calling (MC3) from TCGA were used to identify somatic mutations associated with age. Using logistic regression analysis, we analysed whether age at diagnosis affects somatic mutations in LGG (Table S1) and GBM (Table S2). Results showed that, in patients with LGG, *IDH1*, *ATRX*, and *TP53* were less likely to mutate with aging, while *EGFR* acquired more mutations with aging (*p*-value < 0.05 after Bonferroni correction) (Figure 1a). Similar to LGG, *ATRX* was less likely to mutate with age in GBM (*p*-value < 0.05 after Bonferroni correction) (Figure 1b). Additional mutations information of four genes was included in supplementary Table S3. To investigate the effect of the compounding factor, additional analysis in control models was performed and the results were presented in Supplementary Table S4.

### 2.2. Histological Tumour Grade Depends on Age at Diagnosis in the IDH1 Wild-Type Group but Not in the Mutant Group

**Figure 2:**
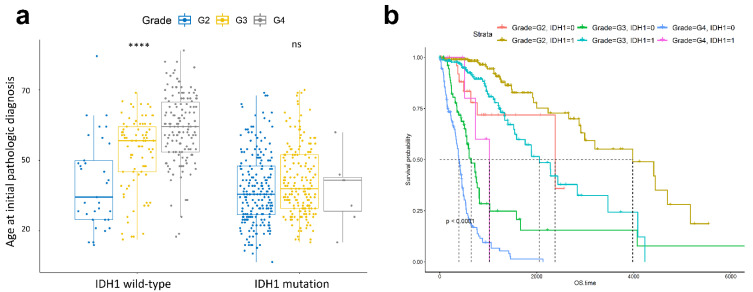
Comparison of tumour grades of patients with *IDH1* wild-type and *IDH1* mutation with respect to age at initial pathologic diagnosis. *p*-value <0.0001 are indicated by ****. ns is not significant (**a**). Survival analysis comparing patients with *IDH1* wild-type or *IDH1* mutation in each tumour grade. 0 indicates wild-type; 1 indicates mutation. Time up to which 50% patients survived is shown by the dotted line in every type. The unit of overall survival (OS) time is day (**b**).

The patterns of age and *IDH1* mutations were analysed according to histological tumour grade. Results showed that, in patients with *IDH1* wild-type, as the histologic tumour grade increased, the onset age of glioma was increased (Figure 2a). However, in *IDH1* mutant patients, despite the rising histologic tumour grade of cancer, the onset age of glioma was constant (Figure 2a). In patients with G2 and G3 tumours, OS differed significantly between patients with and without *IDH1* mutation (log-rank *p*-value in G2: 0.002; in G3: 1E-11; in G4; 0.02) (Figure 2b and Table S5). In particular, the OS of patients with *IDH1* wild-type and LGG did not differ significantly from that of patients with *IDH1* mutation in GBM patients, but median survival was lower (median survival; *IDH1* wild-type in LGG: 758 and *IDH1* mutant in GBM: 1024) (Figure 2b).

### 2.3. Transcriptomic Landscape of Tumours of Different Histological Grades Harbouring IDH1 Mutations

**Figure 3:**
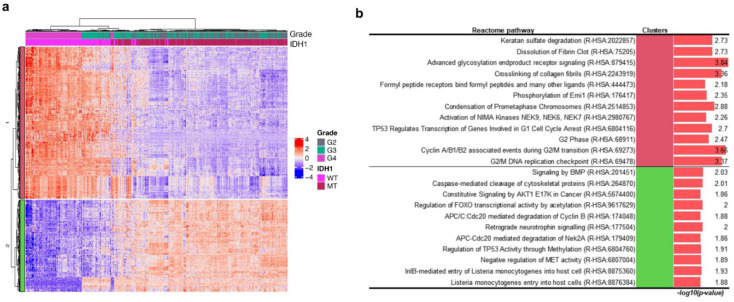
Heatmap with hierarchical tree of patients by gene expression pattern with information gain (IG) over 0.35. The top annotations show tumour grade and mutation status (*IDH1* mutant or wild-type). WT indicates wild-type; MT indicates mutation (**a**). Statistically significant results of the pathway enrichment analysis of gene clusters in the *IDH1* hierarchical group (**b**).

To determine the transcriptomic landscape of tumours of different histological grades harbouring *IDH1* mutation, we performed a multi-label information gain (IG)-based feature selection of genes that possess discriminatory power according to combinations of histological tumour grades and *IDH1* mutation statuses (Table S6). As a result, a hierarchical tree of patients dominantly separated by LGG and GBM was generated (Figure 3a). Interestingly, the gene expression pattern of *IDH1* wild-type of G3 was similar to *IDH1* wild-type of G4. In the *IDH1* wild-type of G4 and G3-rich cluster, genes involved in the mitotic cell cycle and degradation of the extracellular matrix that are associated with malignancy of tumour in the Reactome 2020 database were more highly expressed than in LGG (Figure 3).

### 2.4. Distribution and Prognosis of Age-Dependent Non-Silent Somatic Mutations in LGG

**Figure 4:**
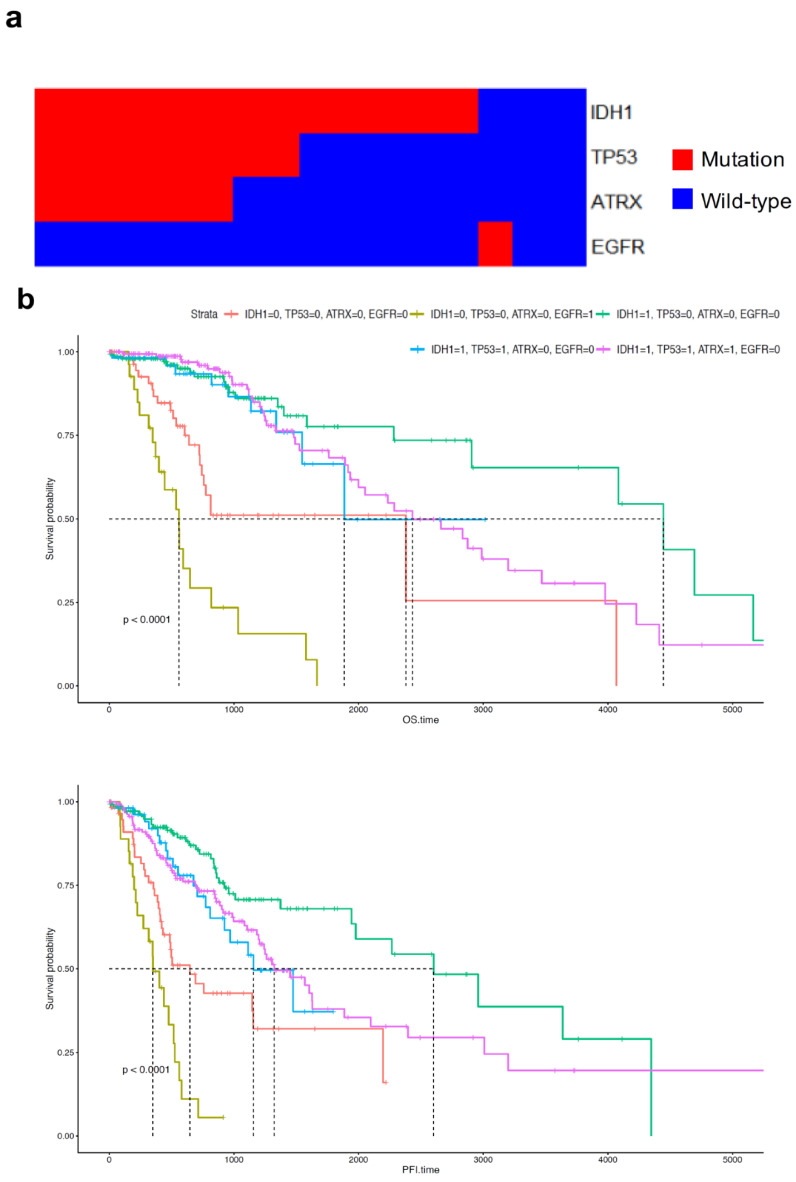
Mutational patterns of *IDH1*, *TP53*, *ATRX,* and *EGFR* in the hierarchical tree (**a**). Survival analysis of patients with mutant or wild-type genes in terms of overall survival and progression-free survival (**b**). 0 indicates wild-type; 1 indicates mutation.

Among several somatic mutations of four genes associated with pathological onset age in LGG, mutation pattern analysis revealed that *ATRX* and *TP53* mutations occurred predominantly in patients with *IDH1* mutations. The *EGFR* mutations dominantly occurred in patients with *IDH1* wild-type (Figure 4a). This analysis included major mutations with over 5 percent incidence among all LGG patients. The proportion of patients divided by mutation status is suggested in supplementary Table S7. The group with *EGFR* mutations had the worst prognosis, in terms of both OS and progression-free interval (PFI) (Figure 4b and Table S8). Among patients with *IDH1* mutations, those with *ATRX* and *TP53* mutations showed poorer PFI than those with the wild-type genes (Figure 4b).

### 2.5. ATRX and TP53 Mutation Occurred in Patients with Early Onset Age of G2 and G3 Glioma and IDH1 Mutation, and the Gene Expression Patterns of Mutant ATRX and TP53 in LGG Differed from Those of Their Wild-Type Counterpart

**Figure 5:**
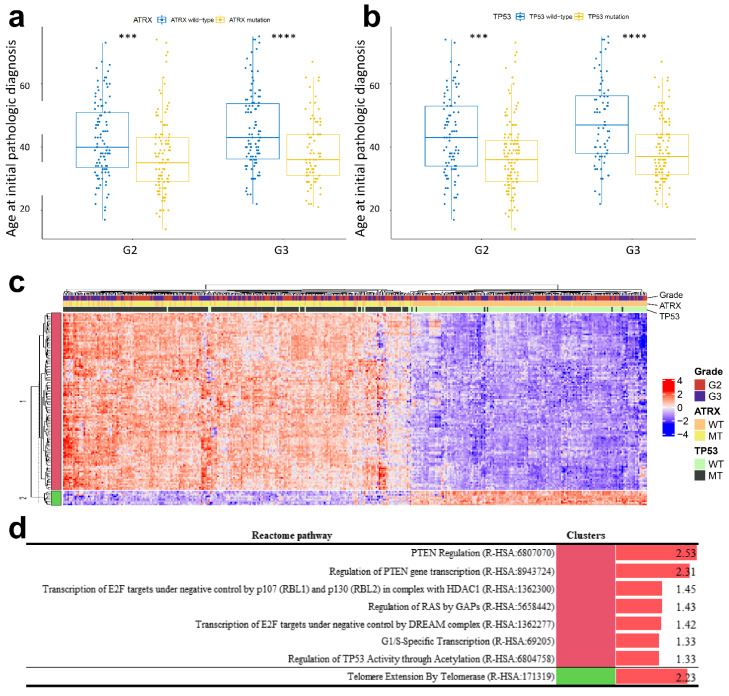
Comparison of patients harbouring *ATRX* and *TP53* wild-type and mutation with age of glioma onset at initial pathological diagnosis in the background of *IDH1* mutation. *p*-values less than 0.001 and 0.0001 are shown by *** and ****, respectively. As the number of glioblastoma (GBM) patients with *IDH1* mutation is few, GBM is excepted in analysis (**a**,**b**). Heatmap with the hierarchical tree of patients by gene expression pattern with information gain (IG) over 0.35. The top annotations show the grade and mutational status (mutant or wild-type) of *ATRX* and *TP53*. WT indicates wild-type; MT indicates mutation (**c**). Statistically significant results of pathway enrichment analysis of gene clusters in the *ATRX* and *TP53* hierarchical group (**d**).

*ATRX* and *TP53* mutations were common in patients with *IDH1* mutation and were associated with the age of onset of LGG (Figure 4a). Therefore, we analysed whether *ATRX* and *TP53* mutations were directly related to the early age of onset of patients with *IDH1* mutation. Results showed that both *ATRX* and *TP53* mutations occurred in patients with early onset age of G2 and G3 tumours (Figure 5a). In the transcriptomic analysis, the hierarchical tree of patients primarily clustered by *ATRX* and *TP53* double mutations and their double wild-type versions rather than by histological grade (Figure 5b). In total, 121 genes passed the IG-based feature selection. Among these 121 genes, the telomere extension-related Reactome term was significantly enriched in the gene enrichment test. This term contained only *TERT* (Figure 5c, Table S9).

### 2.6. Telomerase Reverse Transcriptase (TERT) Expression Was Suppressed in LGG and GBM with ATRX Non-Silent Mutations

**Figure 6:**
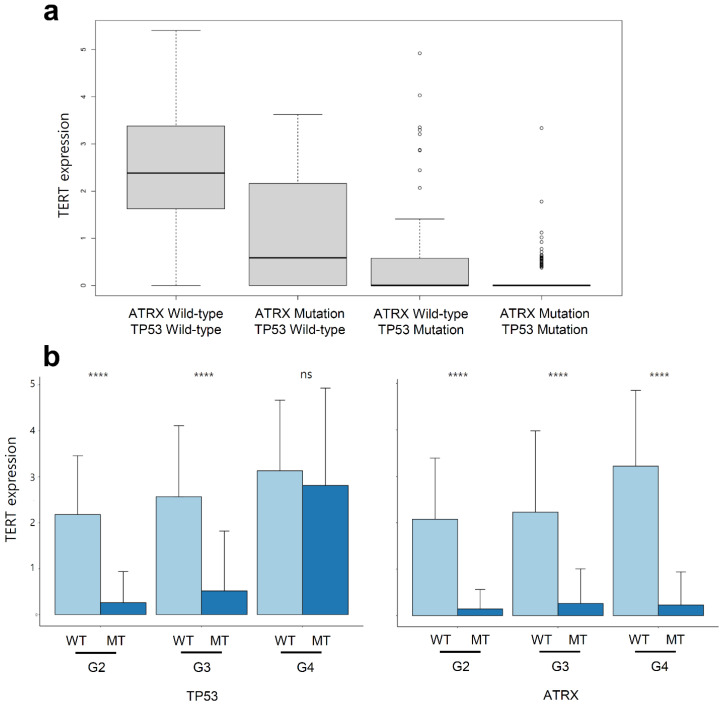
Variation in *TERT* expression level with the mutation statuses of *ATRX* and *TP53* in tumours with *IDH1* mutation (**a**). *TERT* expression level in tumours with *IDH1* mutation with separated mutation statuses of *ATRX* and *TP53* for each grade. WT indicates wild-type; MT indicates mutation. *p*-values < 0.0001 are shown as ****. ns is not significant (**b**).

We observed that *TERT* expression which is only significant in cluster 2 was related to *ATRX* and *TP53* double mutation in the *IDH1* wild-type subgroup in LGG (Figure 5b). In addition, *TERT* expression was highly suppressed in *ATRX* and *TP53* double mutant patients in the *IDH1* mutation subgroup in LGG (Figure 6a). Figure 4a shows that the *ATRX* and *TP53* mutation patterns were similar. Therefore, we compared *TERT* expression with the *ATRX* or *TP53* mutation status and tumour grade. *TERT* expression was highly suppressed in patients with *ATRX* mutation for all histological tumour grades, but not in those with *TP53* mutation of G4 (Figure 6b). It indicated that the *TERT* expression was tightly regulated by *ATRX* but not *TP53* mutation.

### 2.7. EGFR Mutation Occurred at Late Onset Age in Patients with G3 Tumour and Was Associated with Poor Prognosis of GBM Patients but Not Was Not Clustered in Transcriptomic Analysis

**Figure 7:**
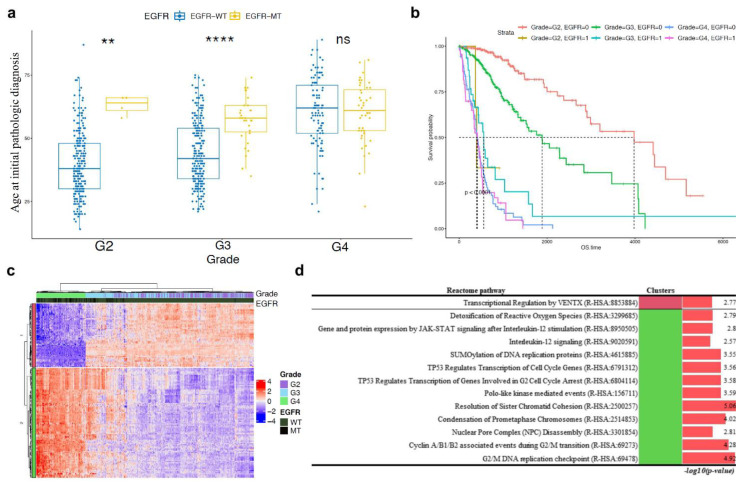
Comparison of ages at initial pathological diagnosis of tumours with *EGFR* wild-type and those with *EGFR*. *p*-values <0.0001 are shown as ****. *p*-values <0.01 are shown as **. ns is not significant (**a**). Survival analysis comparing *EGFR* mutant or wild-type and each grade with overall survival. 0 indicates wild-type; 1 indicates mutation. The unit of overall survival time is day (**b**). Heatmap with hierarchical tree of patients based on gene expression pattern with IG over 0.35. Top annotations show tumour grade and genotype (mutant or wild-type) of *EGFR*. WT indicates wild-type; MT indicates mutation (**c**). Statistically significant results of pathway enrichment analysis of gene clusters in the *EGFR* hierarchical group (**d**).

Unlike *ATRX*, *TP53*, and *IDH1* mutations, *EGFR* mutations were more frequent in older patients. Age at initial pathologic diagnosis of patients with wild-type *EGFR* was positively correlated with tumour grade. Interestingly, in G2 and G3 patients who have relatively early onset age, the patients with *EGFR* mutation had tumour occurrence at old age as well as G4. In addition, despite having LGG, patients with *EGFR* mutations in G3 showed poor prognosis similar to those of GBM patients (Figure 7b). In the transcriptomic analysis, the hierarchical tree of patients was dominantly divided into LGG and GBM, but not by *EGFR* mutation status (Figure 7c, Table S10). Most of the significant pathways in the pathway enrichment analysis were related to the regulation of the cell cycle and were downregulated in GBM (Figure 7d).

## 3. Discussion

Cancer is highly related with the occurrence of somatic mutations, which accumulate with age [13,14]. In this study, we deeply investigated characteristics of biological mechanisms or pathways according to gene expression patterns of age-related somatic mutations in glioma.

In the *IDH1* mutation group, cell-cycle-related genes are down-regulated in contrast to *IDH1* wild-type group (Figure 3a,b). However, hypermethylation of *TP53* occurs in *IDH1* mutant, owing to the accumulation of 2-HG [15]. In contrast, regulation of *TP53* expression via methylation does not occur against the *IDH1* wild-type background. Hypermethylation of DNA in *IDH1* mutants results in the formation of the oncometabolite, 2-HG [16]. In short, most patients of grade 2 and 3 tumours with *IDH1* mutation show suppression of cell growth and high methylation of *TP53*, resulting in the downregulation of *TP53* transcription in younger glioma patients. In contrast, promotion of cell growth and low methylation of *TP53* are observed in most patients of grade 4 and a few of grade 3 tumours with *IDH1* wild-type in older patients. Cell migration Reactome terms were also included in the analysis. In the *IDH1* wild-type background, degradation of keratan sulphate, which can interact with neuroregulatory ligands, dissolution of fibrin clot, and crosslinking of collagen fibrils, which are important for tumour cell and T cell migration, were higher than those in the *IDH1* mutant [17,18,19]. Activation of NIMA kinases, which induce premature mitosis, is highly activated in tumours with *IDH1* wild-type [20,21]. Phosphorylation of early mitotic inhibitor (Emi1), which inhibits the anaphase-promoting complex/cyclosome (APC/C), is necessary for degradation of cyclins in mitosis [22]. APC/C with Cdc20 degrades type B cyclin and Nek2A and facilitates arrest of mitotic division [22,23,24] Activity of Emi is lower for the *IDH1* wild-type background; in contrast, activation of APC/C is higher for the *IDH1* mutant background. In short, mitosis is suppressed more in tumours with *IDH1* mutation than in tumours with *IDH1* wild-type. Advanced glycosylation end product receptor signalling, which regulates cell proliferation, survival, differentiation, migration, and binding of formyl peptide receptors to formyl peptides and many other ligands, thereby regulating angiogenesis, cell proliferation and anti-apoptotic activities, is also higher in tumours with *IDH1* wild-type [25,26]. As a result, negatively regulated MET pathway-related genes were significantly enriched in the *IDH1* mutation-rich cluster (Figure 3b). In cancer, the MET pathway plays an important role in epithelial-mesenchymal transition (EMT) and cancer stemness [27]. In gliomas, the activation of MET promotes the proliferation of tumour and reduces cell death induced by cisplatin, taxol, and gamma irradiation [28]. BMP signalling induces the activation of AKT1 and inhibits cell migration by inhibiting EMT [29]. Retrograde neurotrophin signalling activates PI3K/AKT signalling, which is strongly associated with EMT [30,31]. Foxo1 is a key mediator in ERK2-induced EMT [32]. In the *IDH1* wild-type group, these regulations of EMT-associated terms were down-regulated. The proneuronal, proliferative, and mesenchymal subtypes are identified as WHO grade II and III astrocytomas, and the mesenchymal subtype has a poorer prognosis [33,34].

Unsupervised clustering revealed that mutations in *IDH1*, *TP53*, and *ATRX* occur simultaneously (Figure 4a). Onset of mutations in *TP53* and *ATRX* occur earlier in life, similar to onset of the *IDH1* mutation (Figure 5a,b). In a previous study, *ATRX* and *TP53* mutations with *IDH* mutation were needed in glioma formation in mice model. Genetic changes of primary glioblastoma whose majority cases were IDH wild-type were mutation of *TERT*, *TP53*, and amplification of *EGFR* [35]. Using unsupervised clustering of gene set extracted via multi-label IG, patients were divided on the basis of *TP53* and *ATRX* double mutation and *TP53* and *ATRX* wild-type with *IDH1* mutation (Figure 5c). PTEN regulation, negative control of transcription by E2F, and regulation of *TP53* via acetylation were upregulated in the double mutation group. However, telomere extension by telomerase was promoted in the double wild-type group. Interestingly, the level of *TERT* expression in patients with double mutation was almost zero (Figure 6a). After separating the mutations of *TP53* and *ATRX*, every patient with *ATRX* mutation showed a low level of *TERT* expression; however, *TERT* expression in patients with *TP53* mutation and a grade 4 tumour did not differ from that in the wild-type patients. Recent studies have reported that *TERT* expression prevents extension of telomeres by alternative lengthening of telomeres (ALT), which increases loss of *ATRX*; conversely, decrease in *TERT* expression, which is linked to the hypomethylation of *TERT* promoter, precedes ALT in zebrafish [36,37]. Another study reported that *TERT* expression is downregulated in cells with ALT [38]. In short, *ATRX* loss resulting from *ATRX* mutation induces an increase in ALT and reduces *TERT* expression in hypomethylation of the *TERT* promoter. The lack of *TP53* induces an increase in *TERT* expression [39]. *ATRX* and *TP53* mutations differentially affect *TERT* expression, although we can infer that the impact of *ATRX* mutation is more than that of the *TP53* mutation on prognosis of patients (Figure 6b). Regarding the relationship between *TERT* expression and *ATRX* mutation, we inferred that patients with ALT, which can occur due to loss of *ATRX*, have poorer prognosis than patients who have higher *TERT* expression.

*EGFR* signalling induces proliferation, which is one of the common events in various types of cancer [40]. *EGFR* fusion and deletion variants were presented in nearly half of GBM patients, and these alterations induced differential biological process and/or response to targeted inhibitor [41]. Interestingly, the occurrence of *EGFR* mutation increased with aging and was independent of its occurrence with *IDH1*, *TP53*, and *ATRX* mutations (Figure 1a, Figure 4a and Figure 7c). Unlike in patients with GBM, *EGFR* mutation occurred frequently in older patients with LGG. Patients with LGG harbouring *EGFR* mutation showed poor prognosis for both grade 2 and 3; however, *EGFR* mutation in GBM did not affect prognosis (Figure 7a). After conducting multi-label IG and pathway enrichment analysis, most of the related pathways were found to be involved in the regulation of the cell cycle and cell signalling. Cell-cycle related pathways were downregulated and transcriptional regulation by VENTX was upregulated in grade 3 glioma and GBM. Detoxification of reactive oxygen species and interleukin-12 (IL-12) signalling, which exert an anti-tumour effect on tumours, including glioma and glioblastoma, were downregulated in grade 3 glioma and GBM [42,43,44,45].

## 4. Materials and Methods

### 4.1. Description of TCGA Data and Entire Analysis Process

**Figure 8:**
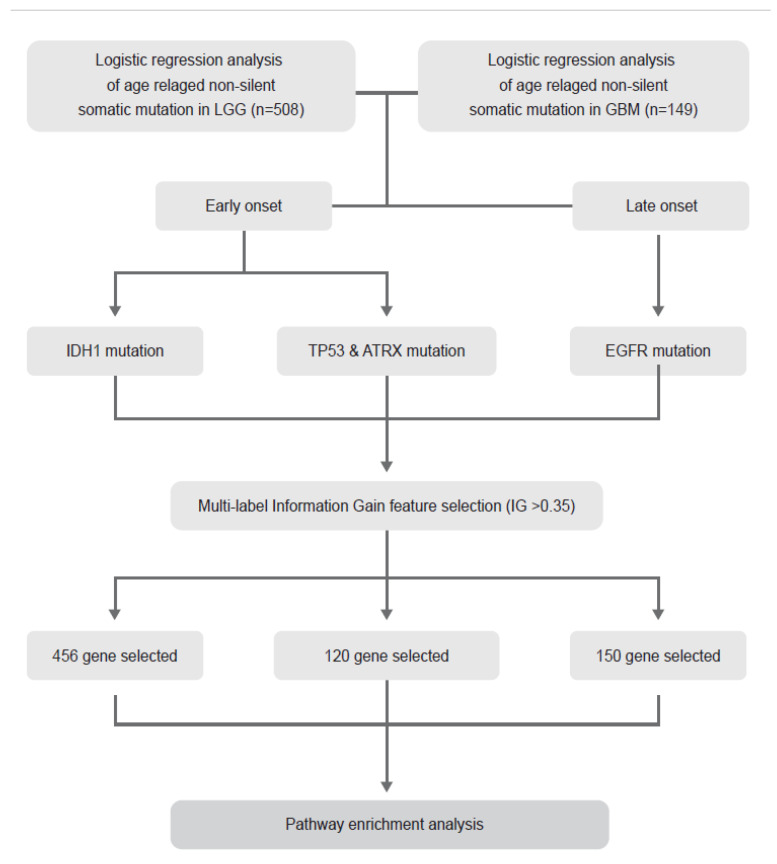
The flow chart of entire process of analysis.

Transcriptome sequencing and clinical data of GBM and LGG were downloaded from the UCSC Xena database. The tumour statuses were LGG (*n* = 508) and GBMLGG (*n* = 657), and all the samples of the GBMLGG dataset were primary tumours (*n* = 657). All samples used in this study have clinical data, survival data, non-silent somatic mutation data, and transcriptomic data. The description of entire analysis process is provided as a flow chart (Figure 8).

### 4.2. Extraction of Candidate Somatic Mutations Related to Age

Candidate somatic mutations related to ages were selected based on a somatic mutation rate of at least 5% from somatic mutation data from multi-centre mutation calling (MC3). Somatic mutation rate is calculated by individual who has mutation/all individuals. Logistic regression analysis was performed to identify somatic mutations related to age. Bonferroni correction was conducted to correct the *p*-value of logistic regression. Logistic regression analysis was conducted by binomial and corrected *p*-value which is 0.05 and 95 percent confidence intervals. The somatic mutation rate and results of logistic regression were visualised using forest plot v 1.10 (a package in R, A Language and Environment for Statistical Computing, R Core Team, R Foundation for Statistical Computing, Vienna, Austria).

### 4.3. Comparing Age with Mutation Status and Grade

The relationship between non-silent somatic mutations selected by logistic regression analysis and onset age are validated by comparing age at initial pathologic diagnosis with each mutation and grade. These results are visualised by ggpubr v 0.4.0 (a package in R, A Language and Environment for Statistical Computing, R Core Team, R Foundation for Statistical Computing, Vienna, Austria).

### 4.4. Survival Analysis

Pairwise log-rank tests with Benjamini-Hochberg corrections were performed to compare prognoses between the groups, which were divided via agglomerative clustering with whole genes; Kaplan-Meier plots were generated in all survival analyses. Survival analysis was conducted using survival v 3.2 (a package in R, A Language and Environment for Statistical Computing, R Core Team, R Foundation for Statistical Computing, Vienna, Austria), and the survival plot was visualised using survminer v 0.4.8 (a package in R, A Language and Environment for Statistical Computing, R Core Team, R Foundation for Statistical Computing, Vienna, Austria), both of which are R packages.

### 4.5. Multi-Label IG Based Feature Selection

Multi-labels, based on the histological grade and presence of somatic mutations in specific genes, were allotted to each individual. IG using multi-label individual and gene-level expression data was performed using FSlector v 0.31 (a package in R, A Language and Environment for Statistical Computing, R Core Team, R Foundation for Statistical Computing, Vienna, Austria) [46,47]. IG more than 0.35 was used as the feature selection threshold for the grade-mutation statuses of *IDH1*, *ATRX*, *TP53*, and *EGFR*.

### 4.6. Hierarchical Clustering and Heatmap with Annotations of Information

Hierarchical clustering of patients was performed by the gene expression levels of the gene set with an IG over 0.35. Visualisation of hierarchical clustering data was performed using a Complex heatmap with annotation, which has information regarding tumour grade and genotype (mutant or wild-type) [48].

### 4.7. Pathway Enrichment Analysis

Reactome pathway enrichment analysis was performed using the ClueGo software, and significant pathways with Benjamini-Hochberg correction < 0.05 were extracted in Cytoscape [49]. *p*-values of the enriched pathways are presented as −log10 (*p*-value).

## 5. Conclusions

Mutations of *IDH1*, *TP53*, and *ATRX* occur in early-onset glioma, whereas *EGFR* mutation is associated with late-onset glioma. Interestingly, the age of patients was increased with histological grade in the presence of wild-type of every gene. In *IDH1* wild-type, patients of G3 and G4 showed similar transcriptomic characteristics involved in mitotic cell cycle. Interestingly, *TP53* and *ATRX* double mutations were predominant in *IDH1* mutation and their prognosis was worse than that of double wild-type. A comparison of the transcriptomic landscape of glioma with wild-type and mutated *IDH1* revealed that the cell cycle- and EMT-related terms were significantly enriched. In *TP53* and *ATRX* double mutation, *TERT* expression was highly suppressed. Suppression of *TERT* expression was caused by *ATRX* mutation. In *EGFR* mutation, hierarchical tree was dominantly separated into LGG and GBM, but *EGFR* mutation status was not. Patients with *EGFR* mutation had the poorest prognosis, whereas those with only *IDH1* mutation had the best prognosis. In *IDH1* mutation, patients with the *TP53* and *ATRX* double mutation showed poorer prognosis than those with the double wild type genotype.

## Data Availability

The data used in this study can find at https://xenabrowser.net/ (accessed on 6 August 2020).

## Supplementary Materials

The following are available online at https://www.mdpi.com/article/10.3390/curroncol28030210/s1, Table S1. The results of logistic regression with gene mutations of which frequency is over 5 percent and age in the lower grade glioma. OR is odds ratio. 2.50% and 97.50% is boundary value of 95 percent confidence interval. Table S2. The results of logistic regression with gene mutations of which frequency is over 5 percent and age in the glioblastoma. OR is odds ratio. 2.50% and 97.50% is boundary value of 95 percent confidence interval. Table S3. The position of mutation. Table S4. Multiple logistic regression analysis between age and age-dependent somatic mutations with adjusted gender and KPS. Table S5. The log-rank *p-value* in Grade and *IDH1* mutation. Grade compares groups of *IDH1* mutation and *IDH1* wild-type in each grade. Mutation compares groups of grades in *IDH1* mutation and *IDH1* wild-type. Table S6. The results of multi-label feature selection information gain of *IDH1* and grade. Table S7. The proportion of patients divided by mutation status. The mutation status information is suggested as *IDH1-TP53-ATRX-EGFR*. WT is wild-type. MT is mutation. Table S8. The log-rank *p*-value in each four age-related mutation. OS is overall survival. PFI is progression free interval. Table S9. The results of multi-label feature selection information gain of *ATRX*, *TP53,* and grade. Table S10. The results of multi-label feature selection information gain of *EGFR* and grade.
