# Supplementary material for: Transcriptomic Landscape of Lower Grade Glioma Based on Age-Related Non-Silent Somatic Mutations
Source: Curr Oncol. 2021 Jun 19;28(3):2281–95. doi: 10.3390/curroncol28030210 (PMC8293196; doi:10.3390/curroncol28030210)
Supplement: Supplementary file 1 [file curroncol-28-00210-s001.zip › Supplementary material.pdf]

Table S1. The results of logistic regression with gene mutations of which frequency is over 5 percent and age in the lower grade glioma. OR is odds ratio. 2.50% and 97.50% is boundary value of 95 percent confidence interval.

Gene	OR	2.50%	97.50%	*p-value*
IDH1	0.95	0.94	0.97	0
TP53	0.95	0.93	0.96	0
ATRX	0.95	0.93	0.96	0
CIC	1.01	1	1.03	0.103
TTN	1.03	1.01	1.05	0.01
FUBP1	1.03	1	1.05	0.017
PIK3CA	1.02	0.99	1.04	0.136
NOTCH1	1.02	1	1.05	0.099
MUC16	1	0.97	1.02	0.82
EGFR	1.1	1.07	1.13	0
NF1	1.03	1.01	1.06	0.019

Table S2. The results of logistic regression with gene mutations of which frequency is over 5 percent and age in the glioblastoma. OR is odds ratio. 2.50% and 97.50% is boundary value of 95 percent confidence interval.

Gene	OR	95% CI (Lower)	95% CI (Upper)	*p-value*
TP53	0.98	0.96	1.01	0.165
PTEN	1.02	0.99	1.05	0.14
EGFR	1	0.97	1.03	0.929
TTN	0.99	0.96	1.01	0.305
MUC16	0.98	0.95	1.02	0.368
SPTA1	1.01	0.98	1.06	0.472
NF1	1	0.96	1.04	0.855
RYR2	0.98	0.95	1.02	0.397
OBSCN	0.95	0.91	0.99	0.013
PIK3CA	0.99	0.95	1.03	0.633
RB1	0.99	0.95	1.03	0.509
FLG	0.98	0.94	1.02	0.253
AHNAK2	0.98	0.94	1.02	0.262
PIK3R1	0.99	0.95	1.03	0.558
PCLO	0.98	0.94	1.02	0.288
MUC17	1	0.96	1.05	0.907
HYDIN	0.98	0.94	1.03	0.458
KEL	1.01	0.97	1.07	0.635
ATRX	0.88	0.82	0.93	0
DNAH17	0.97	0.93	1.02	0.277
DNAH5	0.99	0.95	1.04	0.788
PKHD1	1.01	0.96	1.07	0.649
DOCK5	0.97	0.93	1.02	0.258
COL6A3	1.01	0.96	1.07	0.625
SYNE1	1	0.95	1.06	0.987
GPR98	0.95	0.91	1	0.051
APOB	0.99	0.94	1.04	0.648
CALN1	0.99	0.94	1.05	0.745
SEMA3C	1.01	0.96	1.07	0.589
VWF	0.97	0.92	1.02	0.259
COL1A2	0.97	0.92	1.02	0.17
GRM3	0.97	0.92	1.02	0.27
USH2A	0.96	0.92	1.02	0.154
MROH2B	1	0.95	1.06	0.956
SLIT3	0.97	0.92	1.02	0.179
TCHH	0.98	0.93	1.03	0.348

ARHGEF5	0.95	0.91	1	0.07
RYR3	0.97	0.93	1.03	0.321

Table S4 Multiple logistic regression analysis between age and age-dependent somatic mutations with adjusted gender and KPS

subtype	Gene	Disturbance variable	OR	95% CI (Lower)	95% CI(Upper)	p-value
LGG	IDH1	karnofsky performance score	0.953	0.945	0.961	1.33E-09
		gender	0.992	0.990	0.993	1.34E-09
	ATRX	karnofsky performance score	0.949	0.941	0.957	4.25E-12
		gender	0.949	0.942	0.957	4.02E-12
	TP53	karnofsky performance score	0.947	0.940	0.955	4.15E-14
		gender	0.948	0.941	0.955	5.44E-14
	EGFR	karnofsky performance score	1.099	1.082	1.116	2.36E-12
		gender	1.099	1.082	1.116	2.38E-12
GBM	ATRX	karnofsky performance score	0.876	0.847	0.906	1.58E-07
		gender	0.879	0.851	0.908	6.18E-08

OR; Odds ratio, CI; Confidence interval

Table S5. The log-rank *p-value* in Grade and IDH1 mutation. Grade compares groups of IDH1 mutation and IDH1 wild-type in each grade. Mutation compares groups of grades in IDH1 mutation and IDH1 wild-type

	Grade			Mutation	
	G2	G3	G4	*IDH1* wild-type	*IDH1* mutation
Log-rank *p-value*	0.0023443	9.50E-12	0.015408	3.30E-13	1.95E-08

Table S6. The results of multi-label feature selection information gain of IDH1 and grade

Gene	Clusters
ACAA2	1
ADAM12	1
ALG3	1
ANKRD53	1
ANXA1	1
ANXA2	1
ANXA2P1	1
ANXA2P2	1
ANXA5	1
AQP5	1
ARAP3	1
ARL9	1
ARSD	1
ASF1B	1
ATP5EP2	1
ATP6V1C2	1
AURKA	1
B3GNT5	1
B3GNT7	1
BCL2L12	1
BGN	1
BOLA3	1
BUB1	1
BZW1	1
C11orf63	1
C12orf48	1
C1orf85	1
C2orf28	1
C5orf35	1
C5orf62	1
C6orf115	1
C9orf64	1
CA3	1
CALU	1
CAPZA1	1
CASP3	1
CASP4	1
CASP6	1

CCDC109B	1
CCDC8	1
CCNA2	1
CCNB1	1
CCNB2	1
CCNYL1	1
CD101	1
CD151	1
CD248	1
CD276	1
CD58	1
CD63	1
CD97	1
CDC20	1
CDCA8	1
CDK2	1
CDKN3	1
CENPA	1
CENPK	1
CENPL	1
CEP55	1
CHI3L1	1
CKLF	1
CKS1B	1
CKS2	1
CLIC1	1
CNIH4	1
COL4A1	1
COL5A2	1
DAP	1
DCTD	1
DDB2	1
DDOST	1
DEDD2	1
DEPDC1	1
DMRTA2	1
DPYD	1
E2F7	1
ECT2	1
EDARADD	1
EDEM2	1

EFEMP2	1
EMP3	1
EPR1	1
EVC2	1
F11R	1
FAM109B	1
FAM176B	1
FBXO17	1
FBXO39	1
FKBP9	1
FMOD	1
FSTL1	1
FUCA2	1
G0S2	1
GDF15	1
GGH	1
GJC1	1
GLA	1
GLB1	1
GNG12	1
GPN1	1
GPR1	1
GPX7	1
GPX8	1
GSTK1	1
GUSB	1
HAUS1	1
HDAC3	1
HDHD3	1
HEBP2	1
HEXB	1
HFE	1
HIST1H2BH	1
HJURP	1
HOTAIR	1
HOXA1	1
HOXA2	1
HOXA3	1
HOXA5	1
HOXB3	1
HOXC10	1

IFNGR2	1
IGF2BP3	1
IGFBP2	1
IKBIP	1
ITGA5	1
KDELC2	1
KDELR1	1
KDELR2	1
KDELR3	1
KIAA0101	1
KIAA0495	1
KIF20A	1
KIF23	1
KIF4A	1
KPNA2	1
LAMC1	1
LATS2	1
LDHA	1
LEPRE1	1
LGALS1	1
LGALS3	1
LOC100130776	1
LOC284276	1
LOC407835	1
LOC493754	1
LOC541471	1
LOC647946	1
LOX	1
LOXL1	1
LSP1	1
LYPLA1	1
MAP1LC3C	1
MARVELD1	1
MDK	1
MED8	1
MELK	1
MEOX2	1
METTL7B	1
MIR155HG	1
MLX	1
MMP11	1

MSN	1
MYD88	1
MYL12A	1
NANP	1
NCRNA00152	1
NNMT	1
NOX4	1
NSUN7	1
NTAN1	1
NUP37	1
OCIAD2	1
OSTC	1
OTP	1
PA2G4P4	1
PCNA	1
PCOLCE	1
PDIA3P	1
PDIA4	1
PDIA6	1
PDLIM1	1
PDLIM4	1
PDPN	1
PGCP	1
PION	1
PLA2G2A	1
PLAT	1
PLAUR	1
PLEK2	1
PLP2	1
PMM2	1
PMS2L2	1
POC1A	1
POLR1D	1
POSTN	1
PPCS	1
PPIA	1
PPIC	1
PPIL5	1
PPPDE1	1
PRDX4	1
PRICKLE3	1

PRPS2	1	
PSMC2	1	
PSRC1	1	
PTGFRN	1	
PTTG1	1	
PTX3	1	
PVT1	1	
PYGL	1	
RAB32	1	
RAB34	1	
RAB36	1	
RAB42	1	
RABL5	1	
RAD54B	1	
RAET1K	1	
RAP1B	1	
RARRES2	1	
RARS	1	
RBP1	1	
RFC2	1	
RINL	1	
RPA3	1	
RPS2P32	1	
RRM2	1	
S100A4	1	
SAA1	1	
SDF4	1	
SEC24D	1	
SEC61G	1	
SERPINH1	1	
SH2D4A	1	
SHOX2	1	
SHQ1	1	
SKA1	1	
SLC25A24	1	
SLC27A3	1	
SLC2A10	1	
SLC35A2	1	
SLC43A3	1	
SMC4	1	
SPAG4	1	

SPON2	1
SPRY1	1
STAC	1
STEAP3	1
SUMO1P3	1
TAGLN2	1
TBL2	1
TCEA3	1
TEAD3	1
TGIF1	1
TICAM2	1
TIMP1	1
TMBIM1	1
TMEM106C	1
TMEM159	1
TMEM165	1
TMEM214	1
TMEM60	1
TMEM71	1
TMSB10	1
TMSL3	1
TNFAIP6	1
TNFRSF11B	1
TNFRSF12A	1
TOM1L1	1
TPM4	1
TTC26	1
TTYH3	1
TUBA1C	1
TXNDC5	1
TYMS	1
UBE2A	1
UBE2MP1	1
ULBP3	1
VASN	1
VAV3	1
WEE1	1
XKR8	1
YIPF1	1
ZCCHC9	1
ZNF90	1

AARS	2
ABI1	2
ABLIM1	2
ACBD5	2
ACVR2B	2
AKAP6	2
ALCAM	2
ALDH2	2
ALDH5A1	2
ANKRD16	2
ANKRD26	2
ARPP21	2
ASB13	2
ATP6V1G2	2
ATP9A	2
BEND7	2
BMP2	2
BRD7	2
C10orf4	2
C5orf53	2
CBARA1	2
CDC26	2
CDH20	2
CDHR1	2
CPEB3	2
CRTAC1	2
CSDC2	2
CSMD3	2
CYTH1	2
DDX42	2
DGCR2	2
DHTKD1	2
DIP2C	2
DNAJB12	2
DNAJC12	2
DNM3	2
DSCAML1	2
DST	2
DUSP26	2
DYNC1H1	2
EEF1A1P9	2

FAM133A	2	
FAM171A1	2	
FAM190B	2	
FAM192A	2	
FAM21C	2	
FAM71D	2	
FBXO18	2	
FCHSD2	2	
FLRT1	2	
FOXO3B	2	
FOXO4	2	
FRY	2	
FTHL3	2	
GALNT13	2	
GCSH	2	
GLUD1	2	
GUSBL1	2	
H2AFY2	2	
H3F3C	2	
HDAC4	2	
HDAC5	2	
HERC1	2	
HIPK2	2	
HMX1	2	
HNRNPA3P1	2	
HNRNPH3	2	
HNRNPUL2	2	
JMY	2	
JPH3	2	
JPH4	2	
KCNB1	2	
KCNIP3	2	
KIAA0427	2	
KIAA0430	2	
KIAA1279	2	
LARP4B	2	
LOC254559	2	
LOC339674	2	
LOC341056	2	
LOC399744	2	
LOC442454	2	

LOC643387	2
LOC644936	2
LOC728643	2
LRIT2	2
LRRC37A4	2
MAPT	2
MCF2L2	2
MGEA5	2
MIPOL1	2
MLL	2
MLLT6	2
MTPAP	2
MXI1	2
MYO18A	2
MYST4	2
NACA2	2
NACAP1	2
NALCN	2
NAP1L3	2
NCAM1	2
NCRNA00093	2
NET1	2
NLGN2	2
NOG	2
NRG3	2
NSUN6	2
NTNG2	2
NUMA1	2
OPHN1	2
PABPC5	2
PCDH15	2
PDCD4	2
PHLPP1	2
PHYHIPL	2
PIP4K2B	2
PLCB1	2
PPIAL4C	2
PRLHR	2
RABGAP1	2
RANBP17	2
RASL10A	2

RBM17	2
REPS2	2
RICTOR	2
RPL12	2
RPL13AP3	2
RPL17	2
RPL23P8	2
RPL7	2
RPS27A	2
SCAPER	2
SH3GL2	2
SIRT1	2
SLC25A21	2
SLC6A10P	2
SMC3	2
SMOC1	2
SOX8	2
SPIRE1	2
SPTAN1	2
SSTR1	2
SUFU	2
SYT15	2
TAPT1	2
TARSL2	2
TCEAL2	2
TEF	2
TMOD2	2
TNK2	2
TNRC6C	2
TOM1L2	2
TOP1P2	2
TRIM8	2
TTC3	2
TUB	2
UPF2	2
USP34	2
WAC	2
WBP11P1	2
WDR37	2
X..10357	2
X..653553	2

ZCCHC24	2
ZDHHC22	2
ZMYND11	2
ZNF248	2
ZNF25	2
ZNF33A	2
ZNF638	2
ZRANB1	2

Table S7. The proportion of patients divided by mutation status. The mutation status information is suggested as *IDH1-TP53-ATRX-EGFR*. WT is wild-type. MT is mutation.

WT-WT-WT-WT	MT-WT-WT-WT	MT-MT-WT-WT	MT-MT-MT-WT	WT-WT-WT-MT
64	154	58	171	29
0.13	0.32	0.12	0.36	0.06

Table S8. The log-rank p-value in each four age-related mutation. OS is overall survival. PFI is progression free interval

	IDH1	*TP53*	*ATRX*	*EGFR*
OS	2.82E-18	0.05141	0.066567	2.12E-14
PFI	1.10E-17	0.5545189	0.5479266	1.63E-19

Table S9. The results of multi-label feature selection information gain of *ATRX*, *TP53*, and grade

Gene	Clusters
AKIRIN1	1
ARHGEF10L	1
ASAP3	1
BSDC1	1
BTF3L4	1
C19orf2	1
C1orf109	1
C1orf144	1
C1orf174	1
C2orf67	1
CAPZB	1
CCDC123	1
CCDC146	1

CDC42	1
CMPK1	1
CNN3	1
CSDE1	1
CYB561D1	1
DDOST	1
DDX20	1
DNAJC8	1
DNALI1	1
DOCK7	1
DR1	1
EIF3I	1
EVI5	1
EXOSC10	1
FAM54B	1
FBXO42	1
FOXJ3	1
GDAP2	1
GNAI3	1
GNL2	1
GPATCH1	1
GPBP1L1	1
GTF2B	1
H6PD	1
HDAC1	1
HP1BP3	1
HS3ST1	1
INPP5B	1
KDM1A	1
KDM4A	1
KIAA0467	1
KIAA2013	1
KPNA6	1
LEFTY2	1
LOC148189	1
LRRC41	1
LRRC42	1
LSM14A	1
MFN2	1
MIER1	1
MIIP	1

MTF2	1
NADK	1
NFIA	1
NFYC	1
NRAS	1
NRD1	1
NSUN4	1
OR4N2	1
PABPC4	1
PARS2	1
PGD	1
PHACTR4	1
PHC2	1
PHF13	1
PKN2	1
PNRC2	1
PPP1R8	1
PRPF38A	1
psiTPTE22	1
PSMA5	1
PSMB2	1
RBBP4	1
RLF	1
RPAP2	1
RPF1	1
S100PBP	1
SCP2	1
SERBP1	1
SF3A3	1
SFRS4	1
SNIP1	1
SNRNP40	1
SNX7	1
SRRM1	1
ST7L	1
STX12	1
TCEB3	1
THRAP3	1
TMEM167B	1
TMEM69	1
TRNAU1AP	1

TTC4	1	
TXLNA	1	
TXNDC12	1	
UBA2	1	
UBIAD1	1	
UROD	1	
USP48	1	
UTP11L	1	
VCAM1	1	
WASF2	1	
WDR77	1	
WDR78	1	
X15.Sep	1	
YTHDF2	1	
ZNF362	1	
ZZZ3	1	
C1orf230	2	
C6orf138	2	
CHGB	2	
DRAM1	2	
DRG2	2	
EDA2R	2	
GRPEL2	2	
TERT	2	
TRIM67	2	

Table S10. The results of multi-label feature selection information gain of *EGFR* and grade

Gene	Clusters	
ABLIM1	1	
C10orf4	1	
C5orf53	1	
CDC26	1	
CPEB3	1	
DYNC1H1	1	
EEF1A1P9	1	
FAM171A1	1	
FAM190B	1	
FAM71D	1	
FRY	1	
FTHL3	1	

GCSH	1
GUSBL1	1
H3F3C	1
HERC1	1
HNRNPA3P1	1
HNRNPUL2	1
KIAA0427	1
KIAA0430	1
KIAA1279	1
LARP4B	1
LOC341056	1
LOC442454	1
LOC643387	1
LOC644936	1
LOC728643	1
LRRC37A4	1
MLLT6	1
MYO18A	1
NACA2	1
NACAP1	1
NALCN	1
NUMA1	1
PPIAL4C	1
RICTOR	1
RPL13AP3	1
RPL17	1
RPL23P8	1
SCAPER	1
SLC6A10P	1
SPTAN1	1
TARSL2	1
TEF	1
TMOD2	1
TNRC6C	1
TOM1L2	1
TOP1P2	1
TTC3	1
UPF2	1
WBP11P1	1
X..10357	1
X..653553	1

ZMYND11	1
ZNF33A	1
ZNF638	1
ZRANB1	1
ADAM12	2
ALG3	2
ANXA2P1	2
ARAP3	2
ASF1B	2
ATP5EP2	2
ATP6V1C2	2
AURKA	2
C12orf48	2
C2orf28	2
CALU	2
CAPZA1	2
CASP4	2
CCDC109B	2
CCNB1	2
CCNB2	2
CCNYL1	2
CD58	2
CDCA8	2
CDK2	2
CDKN3	2
CENPA	2
CENPK	2
CENPL	2
CEP55	2
CHI3L1	2
CKLF	2
CLIC1	2
CNIH4	2
COL5A2	2
DDOST	2
DEPDC1	2
EDARADD	2
EDEM2	2
EMP3	2
FKBP9	2
FSTL1	2

GGH	2
GLA	2
GLB1	2
GPN1	2
GPX7	2
GPX8	2
GUSB	2
IFNGR2	2
IGFBP2	2
IKBIP	2
KDELR2	2
KPNA2	2
LDHA	2
LOC100130776	2
LOC407835	2
LOC541471	2
LOX	2
LYPLA1	2
MED8	2
MLX	2
NCRNA00152	2
NNMT	2
NOX4	2
NUP37	2
OSTC	2
PA2G4P4	2
PCNA	2
PDPN	2
PLA2G2A	2
PLEK2	2
PMS2L2	2
PPIA	2
PPIL5	2
PRDX4	2
PSMC2	2
PTTG1	2
RAB32	2
RAB42	2
RAP1B	2
RPA3	2
SEC61G	2

SERPINH1	2	
SHOX2	2	
SHQ1	2	
SLC43A3	2	
SMC4	2	
SUMO1P3	2	
TAGLN2	2	
TBL2	2	
TGIF1	2	
TIMP1	2	
TMEM106C	2	
TMSL3	2	
TNFAIP6	2	
TPM4	2	
TUBA1C	2	
UBE2A	2	
UBE2MP1	2	
WEE1	2	
ZCCHC9	2	
ZNF90	2	
